# First data of statistic and ecological behavior of orthoptera insects in arid region (Southern West of Algeria)

**DOI:** 10.1016/j.dib.2020.105857

**Published:** 2020-06-17

**Authors:** Djamel Brahimi, Lotfi Mesli, Abdelkader Rahmouni, Fatima Zohra Zeggai, Bachari Khaldoun, Redouane Chebout, Mohammed Belbachir

**Affiliations:** a University of Salhi Ahmed, Department of Sciences of Nature and Life Naama, 45000, Algeria; b University of Abou Bakr Belkaid, Faculty of sciences of nature and life and Sciences of the earth and the universe, Department of ecology and environment, Tlemcen, 13000, Algeria; c Department of Chemistry, laboratory of polymer chemistry, University of Oran1 Ahmed Benbella, BPN 1524 El'Menouer, 31000, Oran, Algeria; d Centre de recherche scientifique et technique en analyses physico-chimiques (CRAPC), BP 38Bou-Ismail-RP, 42004, Tipaza, Algeria

**Keywords:** Locust, Naama, Insect, Swarms, *Arid*, Wetland, Invasion, Ecology

## Abstract

The activity developed in vast areas of northwest africa causes serious invasions of different species of orthoptera insect which poses a great danger to agriculture and thus to nutrition of peoples and animals in general. In (Algeria), FAO considers the regions of naama, tindouf, adrar and bechar in Algeria as the theater of signaling of swarms and intervention. In this article, we want to shed light on the peculiarities of this insect (orthoptera), its statistics, its species and the families it belongs to in the arid region called naama (southwestern Algeria). The study of orthoptera in the arid region of naama (southern west of Algeria) conducted at three stations (Mecheria, Ben ammar) and the wetland of (Ain ben khelil) during the period from august 2015 until august 2017 allowed to identify nineteen (19) species divided into two sub-orders ensifera and caelifera. They are divided into five families (Tettigonidae, Gryllidae Pamphagidae, Acrididae, and Pyrgomorphidae). Acrididae family is the largest with five species. Oedipodinae subfamily is the most numerous genera and species it includes four (4) different types genders and seven species (7). The highest diversity index of the shannon-weaver is obtained at the wetland ain ben khelil is 2.12 bits, followed by the station of ben ammar with 2.06 bits and station of mecheria remains in third with 1.89 bit values of fairness are close to one corresponding to populations in balance entered them. The determination of species, genders and families of this population is based on several morphological criteria such as the shape of the pronotum, and the color of membranous wings and the shape of the hind legs.

Specifications Table

**Table:** 

Subject	Environnement, Ecology.
Specific subject area	Environment, Ecology,
Type of data	Table, Image and Figure
How data were acquired	The data presented in this work were acquired from the arid region of naama (southern west of Algeria) conducted at three stations namely (Mecheria, Ben ammar) and the wetland of (Ain ben khelil) during the period from august 2015 until august 2017. The determination of Orthoptera species is based on the Chopard key (1943), and the acridoidea catalog of north west africa of Louveaux, A. & al. (1987) .
Data format	Raw and analyzed
Parameters for data collection	The parameters for this data such as species, genders and families of this population is based on several morphological criteria, the shape of the pronotum, and the color of membranous wings and the shape of the hind legs.
Description of data collection	This data describes that acrididae family is the largest with five species. Oedipodinae subfamily is the most numerous genders and species that include four (4) different types’ genders and seven species (7). Described dataset in this paper provides new idea to understand ecologic and statistic life of different species of orthoptera in arid regions of north west of africa.
Data source location	Republic algerian democratic and popular
Data accessibility	Data are supplied with this article

## Value of the data

• Orthoptera generally live in groups in arid and semi arid regions in africa and have a great effect on agriculture and the environment, which is why there is little work on this family of insects. The data in this article will be informative to statistic and ecological studies of different species of orthoptera in arid region especially naama region (southern west of Algeria).
• This data can give of the researchers chemists, biologists or specialists in insect science additional information to understand the privacy of this family of insects such as the preferred climate, food, the number of species and genders of this insect, the lifespan and its physiology or his physiognomy in general.
• Statistic and ecologic values in this Data can be used as a reference for the world organization of agriculture and nutrition as well as the world health organization to stand up to this insect and reduce its spread.
• After understanding the private life of this insect especially its effect on agriculture and the environment, researchers can use this data to develop their knowledge in the field of orthoptera family and to make this data base as a starting point in their next works.The Data obtained in this work can be effectively applied for all insects mostly of orthoptera in arid region of algeria and africa area.
• Through the different parts of this work can say that the additional values of these data is to limit the preferred region of Orthoptera, the different families of this insect, different species, the effect on the environment, the effect on agriculture and without forgetting the physiology and physiognomy of this insect.The data can be highlighted for further studies in development of better study of orthoptera and another insects in africa region.

## 1 Data description

**Scheme 1:**
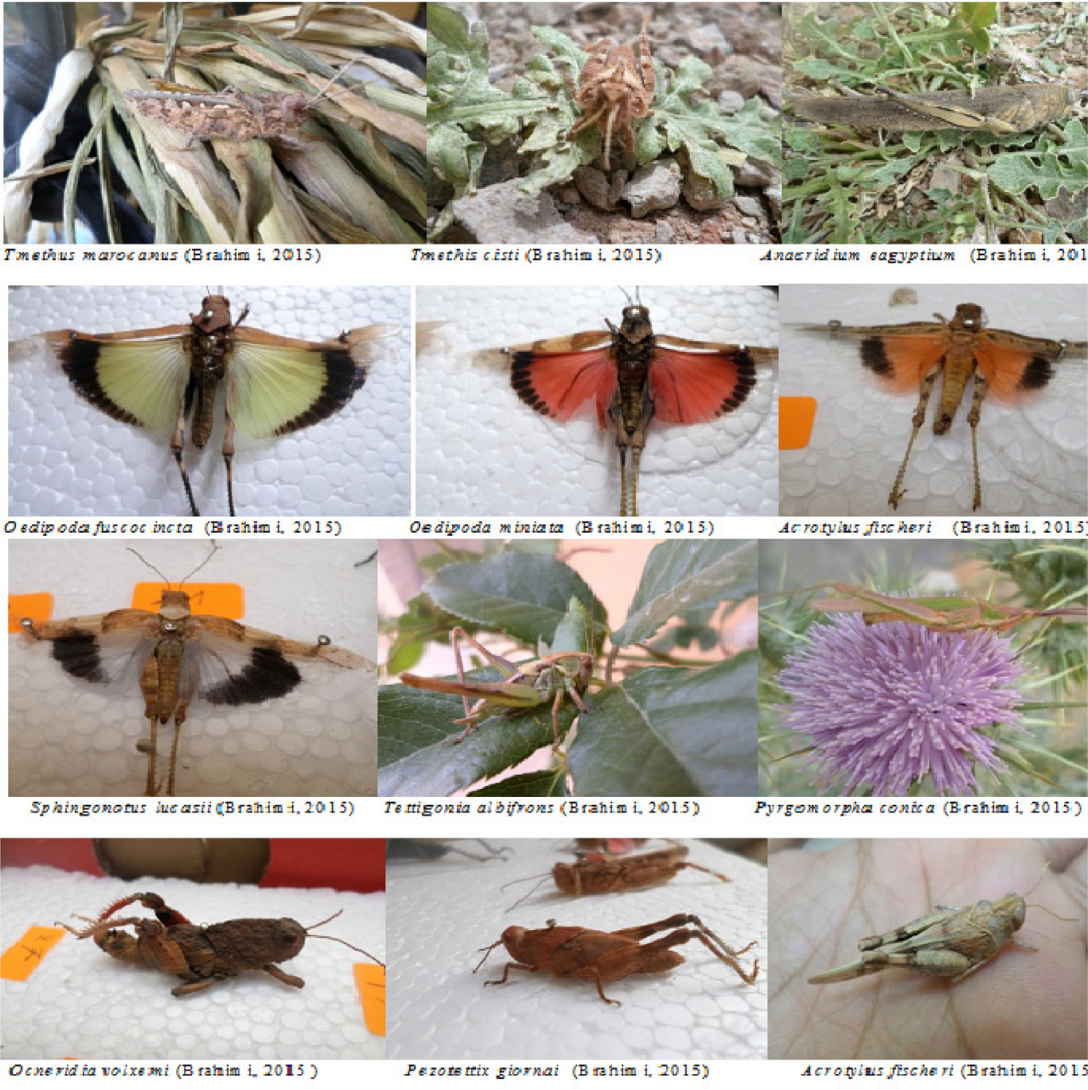
Describes deferent orthoptera species in naama region (southern west of Algeria).

**Fig. 1:**
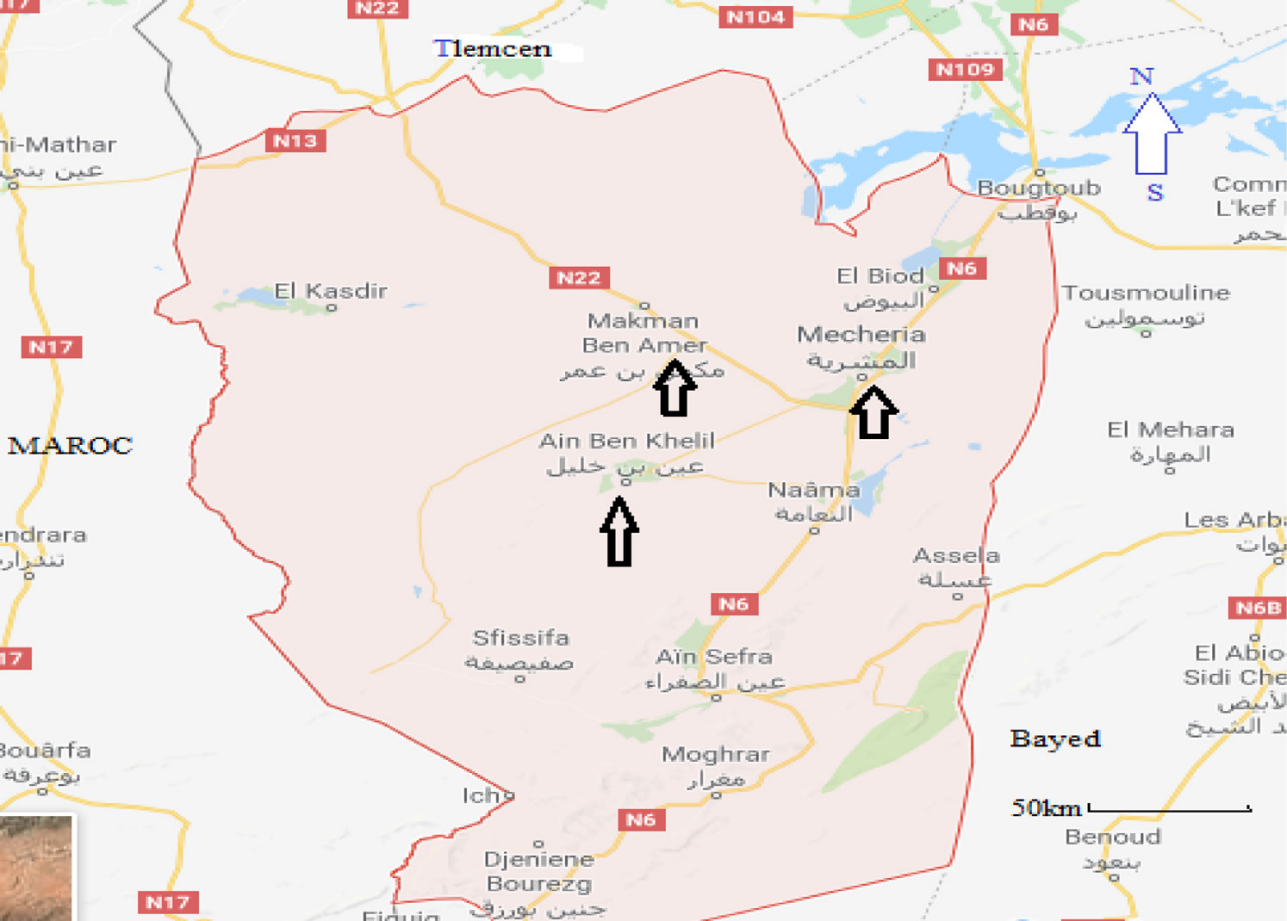
Situation of the three stations in naama region (Algeria).

**Fig. 2:**
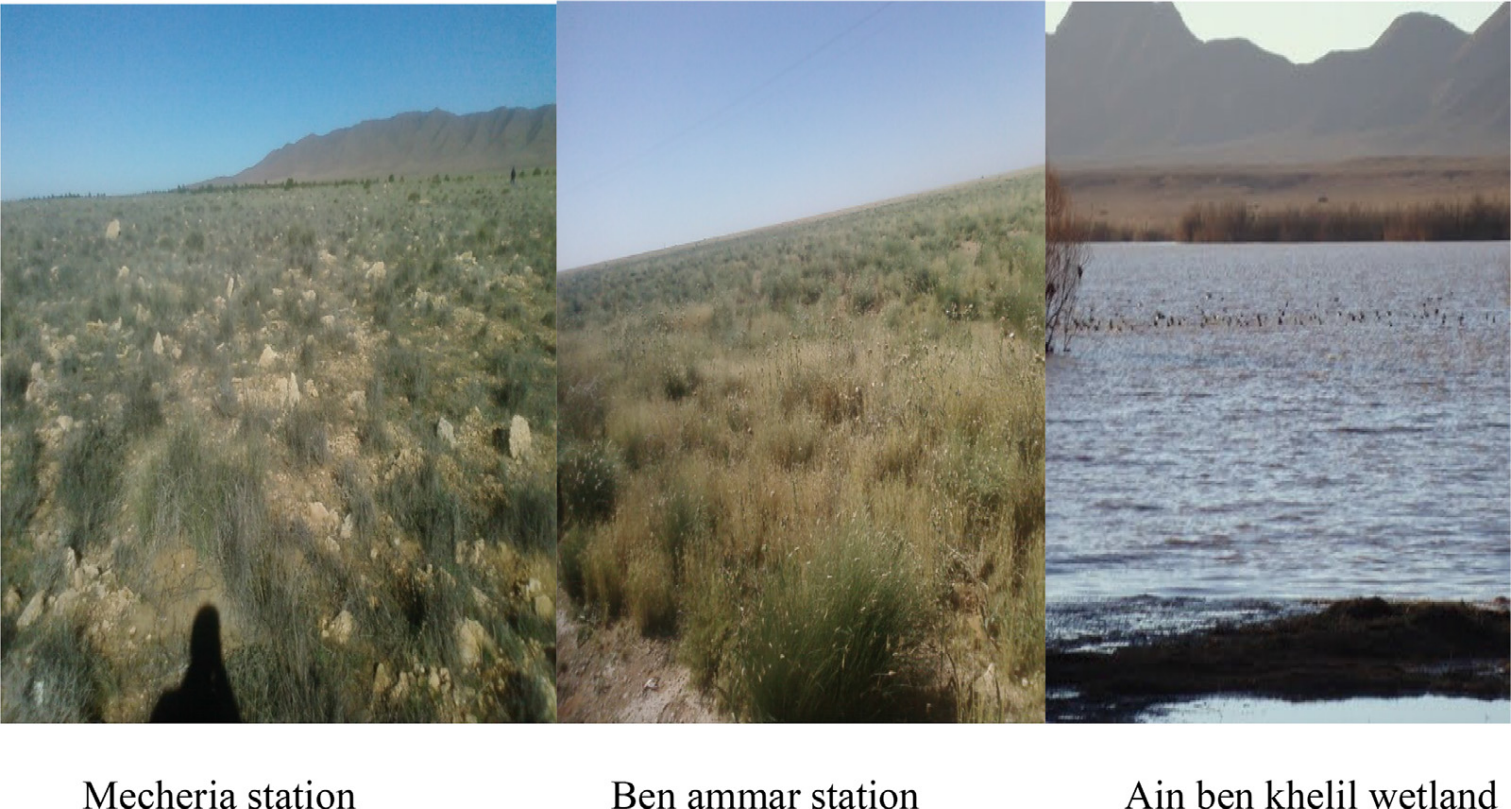
The three stations of the naama region (Southern west of Algeria).

**Fig. 3:**
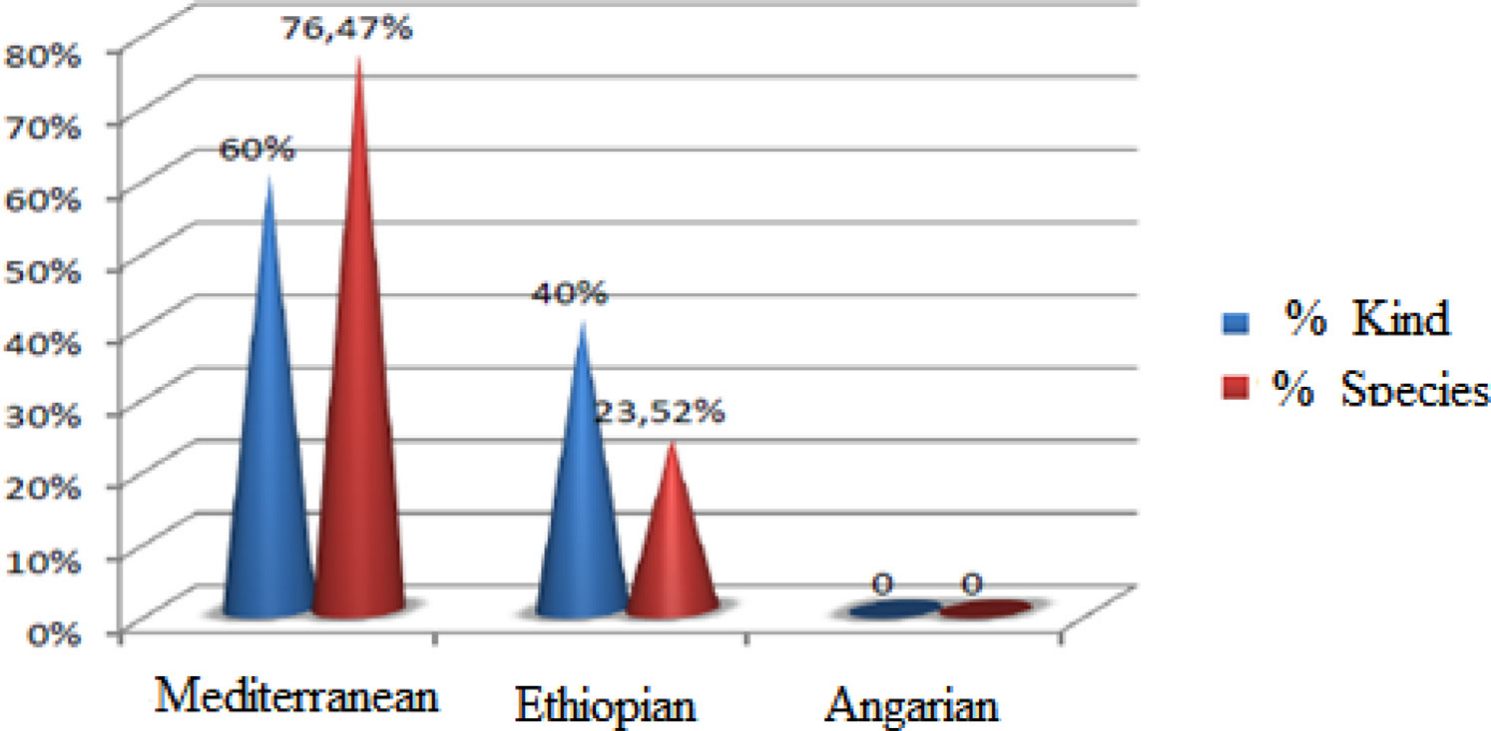
Origin of the locust fauna in the naama region.

**Fig. 4:**
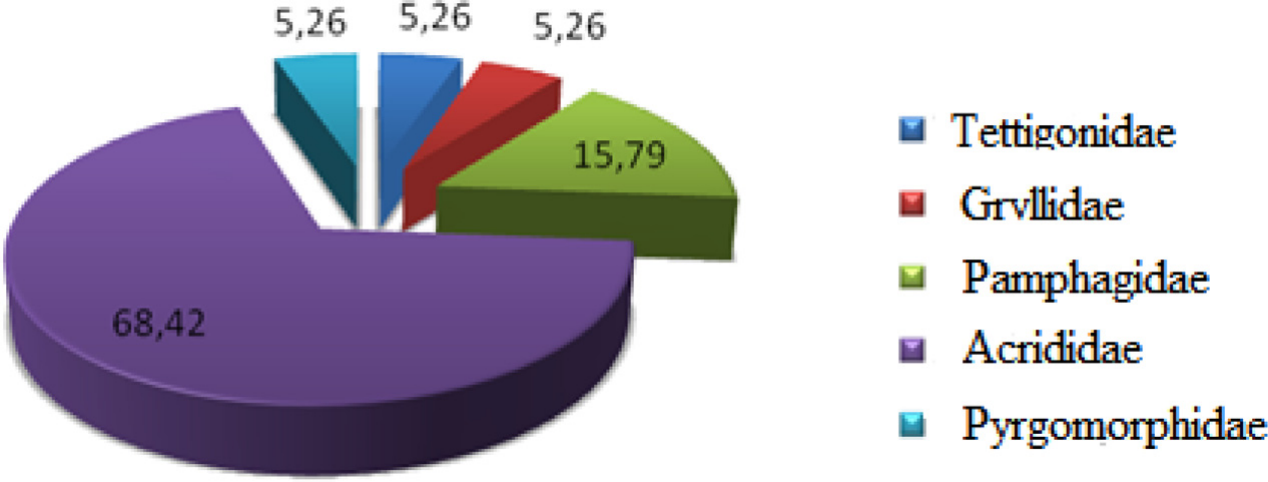
Diversity of the different species of orthoptera family identified in naama region.

**Fig. 5:**
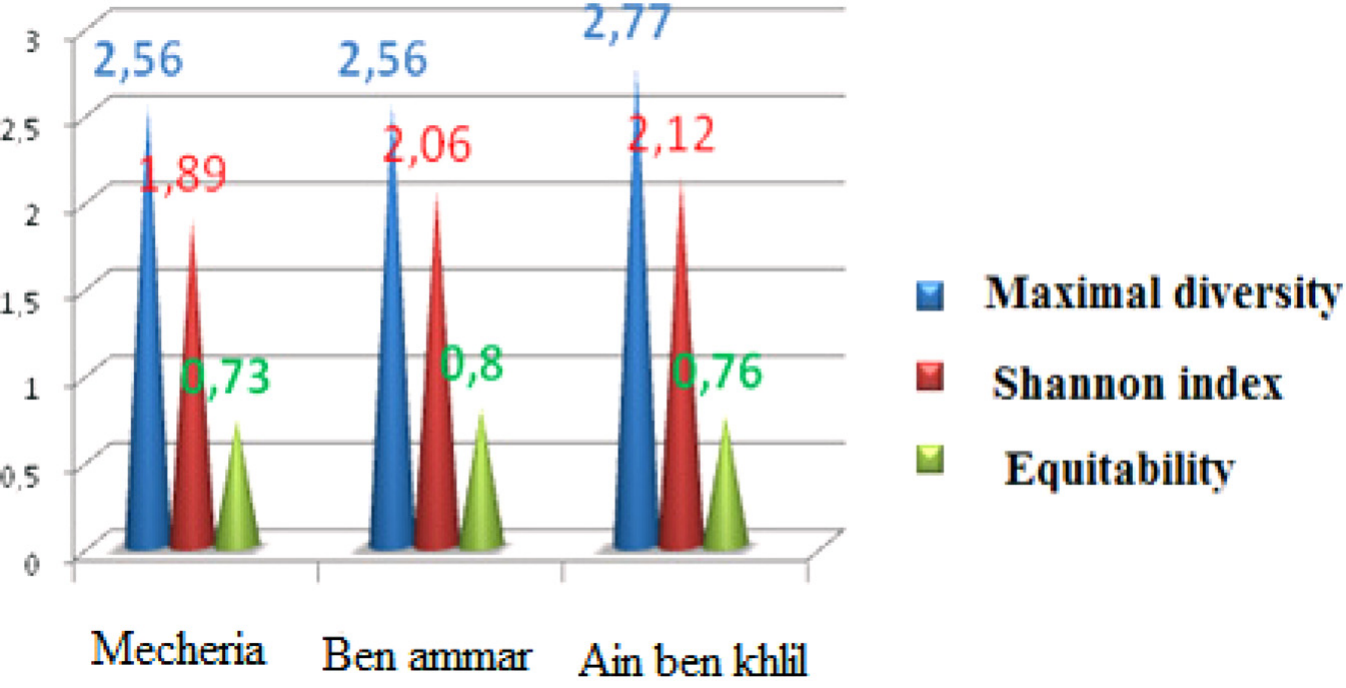
Measurements of maximal diversity, Shannon index and equitability (E) of orthoptera species identified in naama region (Algeria).

**Fig. 6:**
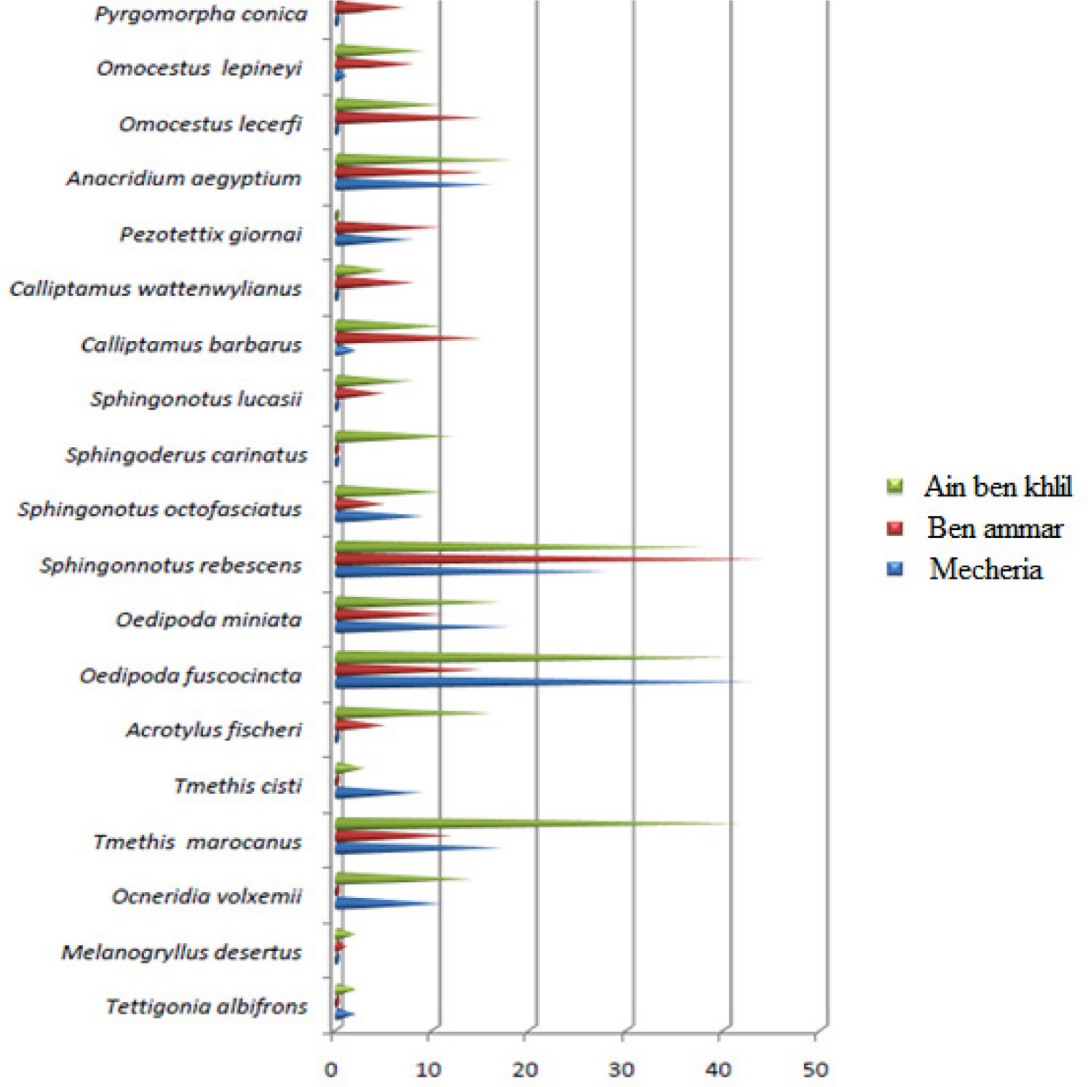
Abundance of species identified in the three stations of naama region.

**Fig. 7:**
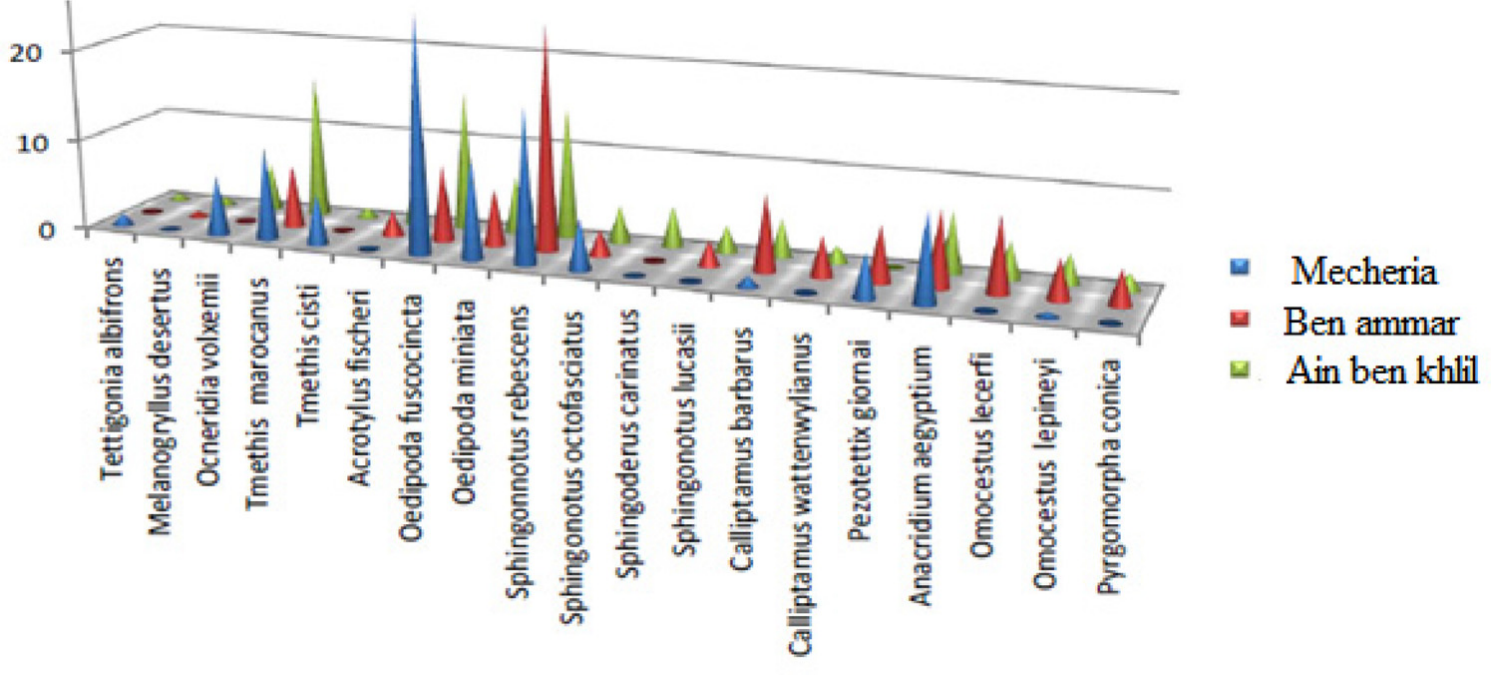
Relative frequencies of orthoptera species identified in naama region.

**Fig. 8:**
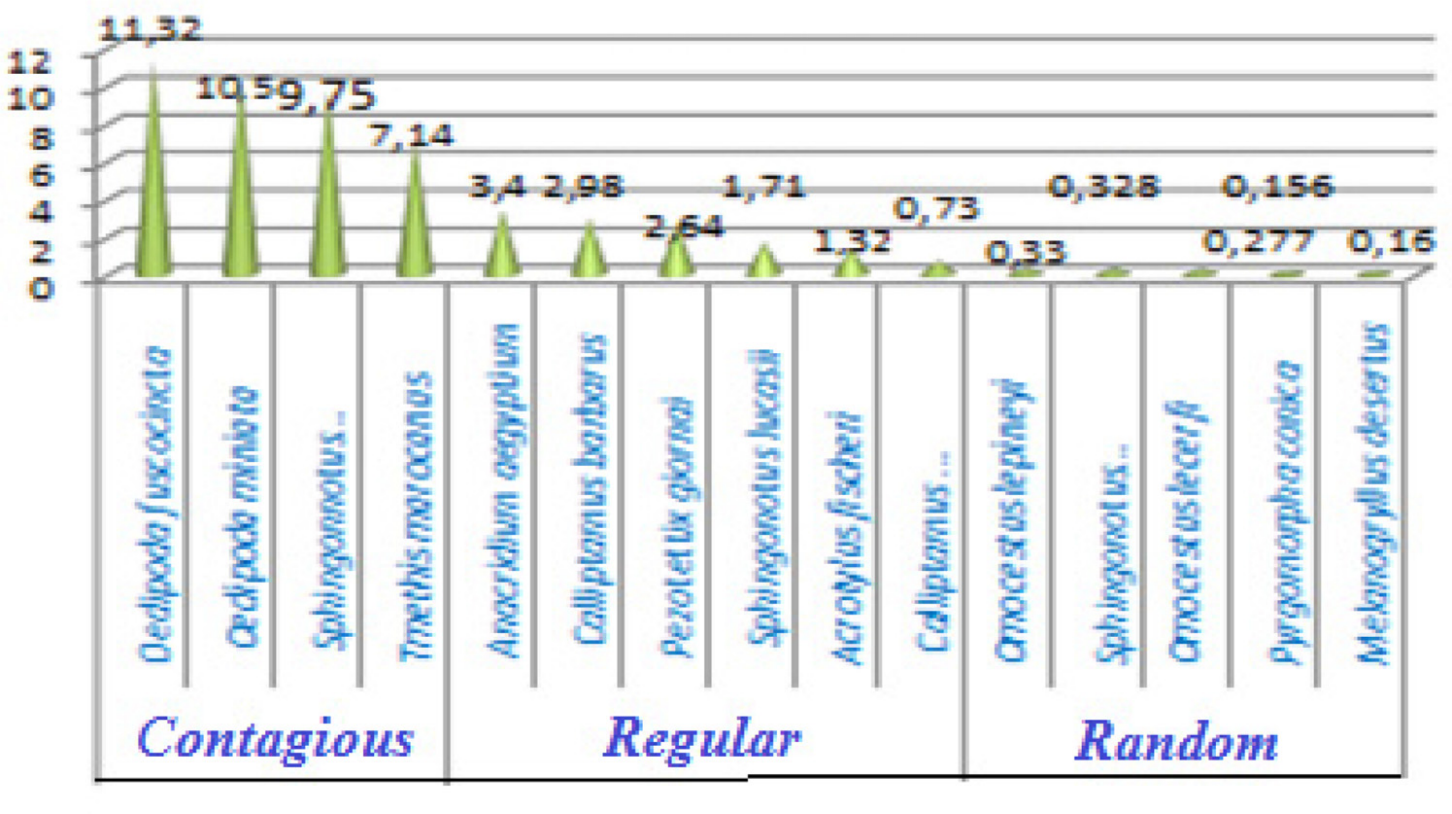
Dispersion index and type of distribution of orthoptera in naama region.

**Fig. 9:**
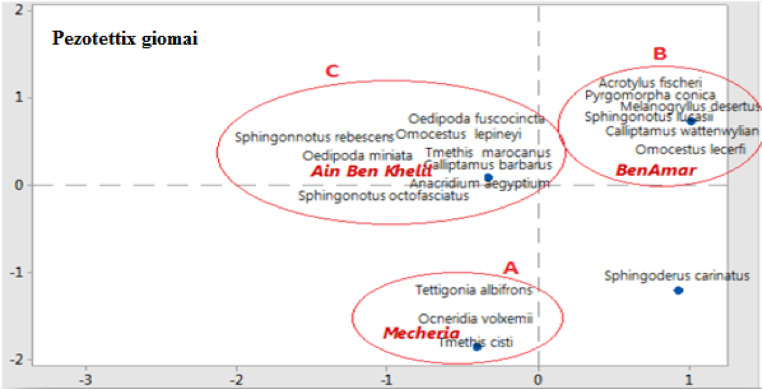
Factorial analysis of the correspondence of orthoptera species of naama region.

**Fig. 10:**
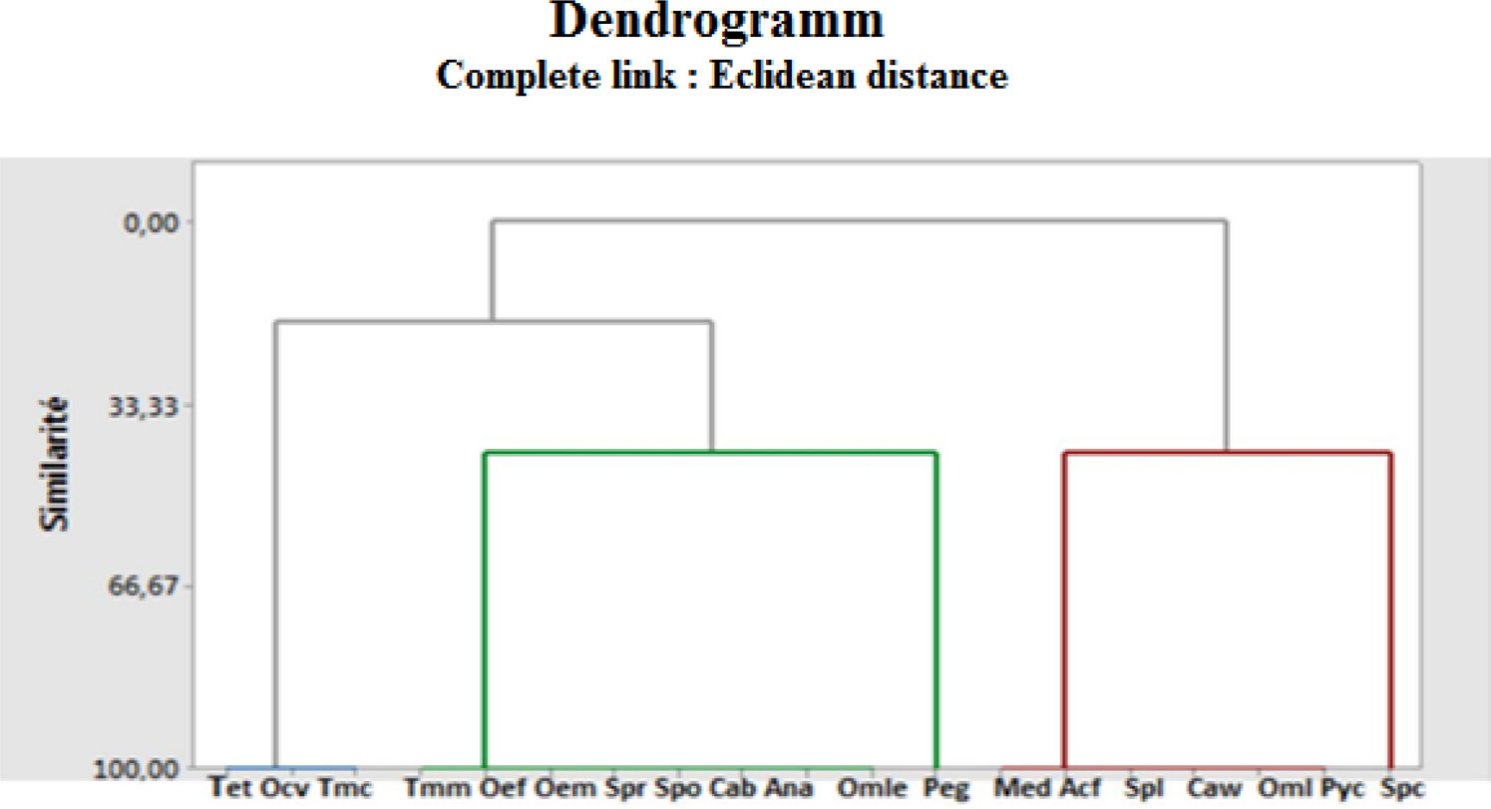
Hierarchical ascending classification of orthoptera species in naama region.

Orthoptera (Locusts) are insects that live in groups such as ants and bees, not alone. They are generally found in arid and semi-arid regions where heat and humidity [1]. Therefore, they are abundant in (Northwest africa). The locusts are found in the form of families consisting of different types and genders, which can be determined by several criteria such as shape, face and color. Locusts play a major role in the environmental balance but it affects the life of the human being by destroying agricultural crops. For this reason, the world food and agriculture organization (FAO) have called on countries to stand up to and combat this insect. In this work the study of locusts or orthoptera for each station of naama region (southern west of Algeria) was based on transects method. Thirteen (13) samples were taken from august 2015 until august 2017.The number of mature individuals belonging to each locust species is counted separately. The collected specimens were preserved by both dry and wet preservation methods [2]. The determination of Orthoptera species is based on the Chopard key (1943), and the acridoidea catalog of north west africa of Louveaux, A. & al. (1987) [3]. Described dataset in this paper provides new idea to understand ecologic and statistic life of different species of orthoptera in arid region.Scheme 1 describes deferent orthoptera species in naama region (southern west of Algeria). Table 1 describes list of species of orthoptera identified in the region of naama (southern west of Algeria). Fig. 1 describes situation of the three stations in the area of naama (Algeria). Fig. 2 describes the three stations of the naama region (South west of Algeria).Fig. 3 describes origin of the locust fauna in the Naama region.Fig. 4 describes diversity of the families of orthoptera species identified in the region of naama. Fig. 5 describes Measurements of maximal diversity, shannon index and equitability (E) of orthoptera species identified in the region of naama (Algeria). Fig. 6 describes abundance of species identified in the three stations of the region of naama. Fig. 7 describes relative frequencies of orthoptera species identified in the region of naama. Fig. 8 describes dispersion index and type of distribution of orthoptera of naama (Algeria). Fig. 9 describes factorial analysis of the correspondence of orthoptera species of naama (Algeria). Fig. 10 describes hierarchical ascending classification of Orthoptera species of naama (Algeria).

**Table 1:** List of species of Orthoptera identified in the region of naama.

sub-order	family	Sub- Family	Genus-species
Ensifera	Tettigonidae	Tettigoniinae	*Tettigonia albifrons*
	Gryllidae	Gryllinae	*Melanogryllus desertus (*Pallas, 1771)
Caelifera	Pamphagidae	Pamphaginae	*Ocneridia volxemii* (Bolivar 1878)
		Thrinchinae	*Tmethis marocanus* (Bolivar 1878) *Tmethis cisti* (Fabricius,1787)
	Acrididae	Oedipodinae	*Acrotylus fischeri* (Azam, 1901) *Oedipoda fuscocincta* (Lucas, 1849) *Oedipoda miniata* (Pallas, 1771) *Sphingonnotus rebescens* (Walker, 1870) *Sphingonotus octofasciatus* (Serville, 1838) *Sphingoderus carinatus* (Saussure, 1888) *Sphingonotus lucasii (*Saussure, 1888)
		Calliptaminae	*Calliptamus barbarus* (Costa, 1836) *Calliptamus wattenwylianus* (Pantel, 1896)
		Catantopinae	*Pezotettix giornai* (Rossi, 1794)
		Cyrtacanthacridine	*Anacridium aegyptium* (Linné, 1764)
		Gomphocerinae	*Omocestus lepineyi (*Chopard, 1937) *Omocestus lecerfi* (Chopard 1936*)*
	Pyrgomorphidae	Pyrgomorphidae	*Pyrgomorpha conica* (Olivier, 1791)

## 2 Materials and methods

### 2.2 Origin of the Locust Fauna of the naama region

During two years of research in the different stations of this arid region, we have succeeded in limiting the different genus of this insect according to the following order:

  - Mediterranean Genus: They occupy the entire mediterranean area (oedipodae calliptamus, sphingonotus, tmethis, omocestus, and ocneridia) [4].
  - Ethiopians Genus: Acrotylus, Anacridium, Pezotettix and Pyrgomorpha.
  - Angariens Genus: any genus of angariens origin in our sample as show in (Fig. 3).

### 2.3 Repartition of Orthoptera species by families

In this work we found five families of orthoptera (tettigonidae, gryllidae, pamphagidae, acrididae and pyrgomorphidae) all species of these families belong to the two sub-order of Orthoptera (Caelifera and Ensifera) as show (Fig. 4) [5].

### 2.4 Study and analysis of the structure of the fauna of Naama region

The number of the species which we inventoried in the region of Naama adds up nineteen (19) species, thirteen (13) species are recorded in both stations mecheria and ben ammar and sixteen (16) species for wetland of ain ben khelil [6]. The value of the shannon weaver diversity index for species caught is 1.89 bit for mecheria, 2.06 bits for ben ammar station and 2.12 bits for the wetland of ain ben khelil. The values of equitability (E) for each station are respectively 0.73 for mecheria station, 0.8 for ben ammar station, and 0.76 in wetland of ain ben khelil. Measurements of maximal diversity show that both mecheria and ben ammar stations represent an equal diversity of 2.66, whereas the high diversity registered in ain ben khelil with 2.77 as show in (Fig. 5).

### 2.5 Quality and sampling effort

Sampling qualities registered at the mecheria station are (0.006), for ben ammar station (0.005) and zero (0) for wetland of ain ben khelil .The three (Q) values tend to zero (0), sampling can be qualified good in the three stations. The gleason score ranges from 5.42 in mecheria station and 5.53 in ben ammar, reaching (6.19) in ain ben khelil wetland, and three values are relatively similar, show that diversity is important [7].

### 2.6 Abundance of Orthoptera species identified in naama region (Algeria)

The total number of individuals of identified in the three stations of the region of naama (Algeria) is six hundred six (606) individuals, the most important abundance values are recorded in the wetland of ain ben khelil (Oedipoda fuscocincta, sphingonnotus rebescens, tmethis marocanus, anacridium aegyptium and oedipoda miniata) are the most abundant species in naama region as show in (Fig. 6) [8].

### 2.7 Relative frequencies of orthoptera species identified in the naama region

At the mecheria station, the highest frequency is that of oedipoda fuscocincta with 26.21%, followed by sphingonnotus rebescens with 17.07%, the study of the frequency of each species in the ben ammar stations allowed to know the highest frequency of the species sphingonnotus rebescens with 24.85% in the ain ben khelil wetland station, the highest frequencies are recorded with the two species tmethis marocanus 15.84% and oedipoda fuscocincta with 15.47% as show in (Fig. 7) [9].

### 2.8 Dispersion index and type of distribution

Tmethis, oedipoda fuscocincta and oedipoda miniata are among the common infected species found in all stations. On the other hand, regular and common species are calliptamus, pezotettix, sphingonotus, acrotylus and calliptamus wattenwylianus as show in (Fig. 8).

### 2.9 Correspondence factor analysis (CFA)

The content present in (Table 1) corresponding to twenteen (20) surveys show the presence of different species in the stations according to the type of environment such as degraded and stony (mecheria station), stepped (ben ammar station) and diversified steppe and rich (wetland). An AFC conducted on this matrix allowed to build a hierarchical classification calculated from the coordinates of species. Dendrogram clearly differentiates three groups of species of unequal size as show in (Fig. 1) [10]:

Group A: It includes species specific to degrade and rocky environments (Mecheria).
Group B: It is mainly represented in the stepped station (Ben ammar).
Group C: it includes species represented in the diversified environments (Ain ben khelil).

The first entity in the right of the projection is the largest as it includes 42.10% of species (8 species). It represents the species caught in wetland of ain ben khelil (Oedipoda fuscocincta, sphingonnotus, oedipoda miniata, omocestus, tmethis, calliptamus, anacridium and sphingonotus octofasciatus).The second entity located in the right of the projection includes six 6 species which are found in the steppe station of ben ammar (Pyrgomorpha, melanogryllus, acrotylus, sphingonotus and calliptamus). The third entity brings together the rest of the species as show in (Fig. 9).

### 2.10 The ascending hierarchical classification (C.H.A)

From the euclidean distances based on the scores of the three factors A.F.C in (Fig. 9), it is possible to recognize three groups. The first includes the surveys carried out in the rock station during the whole year, the second group includes wetland surveys conducted in all seasons and the third concerned with the steppe surveys of ben ammar as show in (Fig. 10).

## Declaration of Competing Interest

The authors declare that they have no conflict of interest.

## Appendix Supplementary materials

Image, application 1
